# Supplementary material for: Characterisation of Streptococcus suis Isolates in the Czech Republic Collected from Diseased Pigs in the Years 2018–2022
Source: Pathogens. 2022 Dec 20;12(1):5. doi: 10.3390/pathogens12010005 (PMC9862946; doi:10.3390/pathogens12010005)
Supplement: Supplementary file 1 [file pathogens-12-00005-s001.zip › Supplementary_Table_S2.pdf]

Table S2: New alleles identified in *S. suis* MLST genes

aroA	cpn60	dpr	gki	mutS	recA	thrA
432	571	385	498	492	355	380
433	572	386	499	493	356	381
434	573	387	500	494		382
435	574	388	501	495		383
436	575	389	502	496		
437		390	503	497		
438		391		498		
				499		
				500		
				501		
