# Supplementary material for: Characterisation of Streptococcus suis Isolates in the Czech Republic Collected from Diseased Pigs in the Years 2018–2022
Source: Pathogens. 2022 Dec 20;12(1):5. doi: 10.3390/pathogens12010005 (PMC9862946; doi:10.3390/pathogens12010005)
Supplement: Supplementary file 1 [file pathogens-12-00005-s001.zip › Supplementary_Table_S3.pdf]

Table S3: New STs identified

ST	aroA	cpn60	dpr	gki	mutS	recA	thrA
2068	94	63	272	106	48	141	28
2069	33	99	293	106	433	26	12
2070	365	3	5	396	28	21	4
2071	38	10	19	26	5	32	27
2073	432	1	1	1	1	1	1
2074	1	6	385	20	28	7	4
2075	38	566	387	503	498	26	28
2076	38	566	40	503	498	26	28
2077	62	296	271	16	495	42	34
2078	147	240	170	95	496	159	98
2079	48	28	49	6	494	26	44
2080	18	572	52	503	489	194	11
2081	229	571	88	197	493	355	62
2082	435	85	72	197	145	42	62
2083	37	152	14	26	8	32	179
2084	35	36	27	13	37	28	29
2085	35	36	27	140	37	28	29
2086	242	308	40	7	98	15	18
2087	434	38	72	197	493	42	62
2088	31	7	109	13	6	7	382
2089	272	573	250	278	492	198	194
2090	18	405	44	7	16	6	380
2091	141	58	132	235	159	282	261
2092	63	75	30	499	22	30	8
2093	31	6	18	20	28	7	4
2094	197	6	18	20	28	7	4
2095	210	575	29	358	34	259	4
2096	13	574	388	501	499	37	117
2097	438	6	18	20	28	7	4

2098	18	572	184	15	489	194	383
2099	18	1	391	12	1	10	25
